# Time to intubate with an innovative intubation device: a dual-center randomized crossover non-inferiority simulation study

**DOI:** 10.1186/s12245-026-01208-y

**Published:** 2026-04-11

**Authors:** Hans Magnus Haveraaen, Udo Seel, Broder Romeike, Sebastian Zinn, Haitham Mutlak, Kai Zacharowski, Vanessa Neef, Benjamin Friedrichson, Jan Andreas Kloka

**Affiliations:** 1 Department of Anesthesiology, Intensive Care Medicine and Pain Therapy, Goethe University Frankfurt, University Hospital, Theodor-Stern Kai 7, 60590 Frankfurt, Germany; 2 Department of Anesthesiology, Intensive Care Medicine and Pain Therapy, Sana Klinikum Offenbach, Offenbach, Germany; 3 Department of Anesthesiology, Columbia University Medical Center, New York City, NY USA

**Keywords:** Endotracheal intubation, Airway management, Simulation study, Emergency medicine, Patient safety

## Abstract

**Background:**

Endotracheal intubation is a high-stakes intervention in emergency airway management, with patient safety closely linked to first-pass success and avoidance of repeated attempts. The Frankfurt Intubation Device (FID) is a bougie-inspired intubation device intended to provide fast protection against aspiration once positioned in the trachea.

**Methods:**

This dual-center randomized crossover simulation study used an easy-to-intubate airway manikin. Ninety participants were divided into three groups of different experience (anesthesiologists, non-physician critical care staff, and medical students) to perform intubation with the Frankfurt Intubation Device (FID) and a standard endotracheal tube with stylet in randomized order. The primary outcome was non-inferiority of time to airway protection, measured from laryngoscope insertion to inflation of a cuffed device positioned in the trachea (T2; Δ = 5 s). Secondary outcomes included first-pass success, tooth damage, and exploratory assessment of training effects, with additional time-based outcomes assessed descriptively.

**Results:**

Across all participants, the FID was non-inferior to the endotracheal tube with stylet for T2 (mean paired difference − 0.77 s, 95% CI − 2.50 to 0.97; non-inferiority margin 5 s). Exploratory analyses demonstrated consistent non-inferiority for T2 across experience groups (G1–G3) and randomization sequences. First-pass success was high in both arms (FID 96.7% vs. ET+stylet 94.4%), while tooth damage occurred less frequently with the FID (7.8% vs. 14.4%). Training effects were observed in the simulator.

**Conclusion:**

In this standardized simulation study using an easy-to-intubate airway manikin, the Frankfurt Intubation Device demonstrated non-inferiority to conventional endotracheal intubation with stylet for time to airway protection. Secondary findings support continued staged evaluation of the FID, particularly in more challenging airway scenarios and among users with limited airway experience, where differences in procedural performance may become more apparent.

**Trial registration:**

Not applicable (simulation study).

**Supplementary Information:**

The online version contains supplementary material available at 10.1186/s12245-026-01208-y.

## Background

Endotracheal intubation (ETI) is a potentially life-saving intervention in emergency medicine and critical care, yet it remains technically demanding and associated with clinically relevant variability in performance. Many clinicians outside anesthesiology perform endotracheal intubation infrequently in routine practice, which may limit skill acquisition and contribute to reduced first-pass success (FPS) in emergency situations. This is relevant because FPS is closely linked to patient safety, while repeated attempts are associated with higher complication rates [1–3]. 

Importantly, challenges in emergency airway management are not confined to inexperienced operators. Critically ill patients, time pressure, and environmental constraints can impair laryngoscopy and limit glottic visualization even among trained providers; adverse peri-intubation events remain common across settings [4–6]. 

Bougie-assisted intubation has gained prominence as a simple, low-threshold strategy that can improve tube placement when only a partial glottic view is achieved [7–9]. Building on this concept, the Frankfurt Intubation Device (FID) combines bougie-guided tube delivery with early mechanical aspiration protection once positioned in the trachea. In an initial user-centered development and evaluation study, the FID demonstrated high usability, a short learning curve, and consistent procedural performance among novice users [10]. 

Before evaluating performance in complex airway scenarios, novel airway devices should be assessed under standardized conditions. The aim of this dual-center, randomized crossover simulation study was to evaluate whether the FID is non-inferior to standard-of-care ETI, using an endotracheal tube with stylet with respect to time to airway protection in an easy-to-intubate airway model. This proof-of-concept design was chosen as a staged step in device development prior to evaluation in more challenging airway conditions and clinical environments.

## Materials and methods

### Study design and setting

**Fig. 1:**
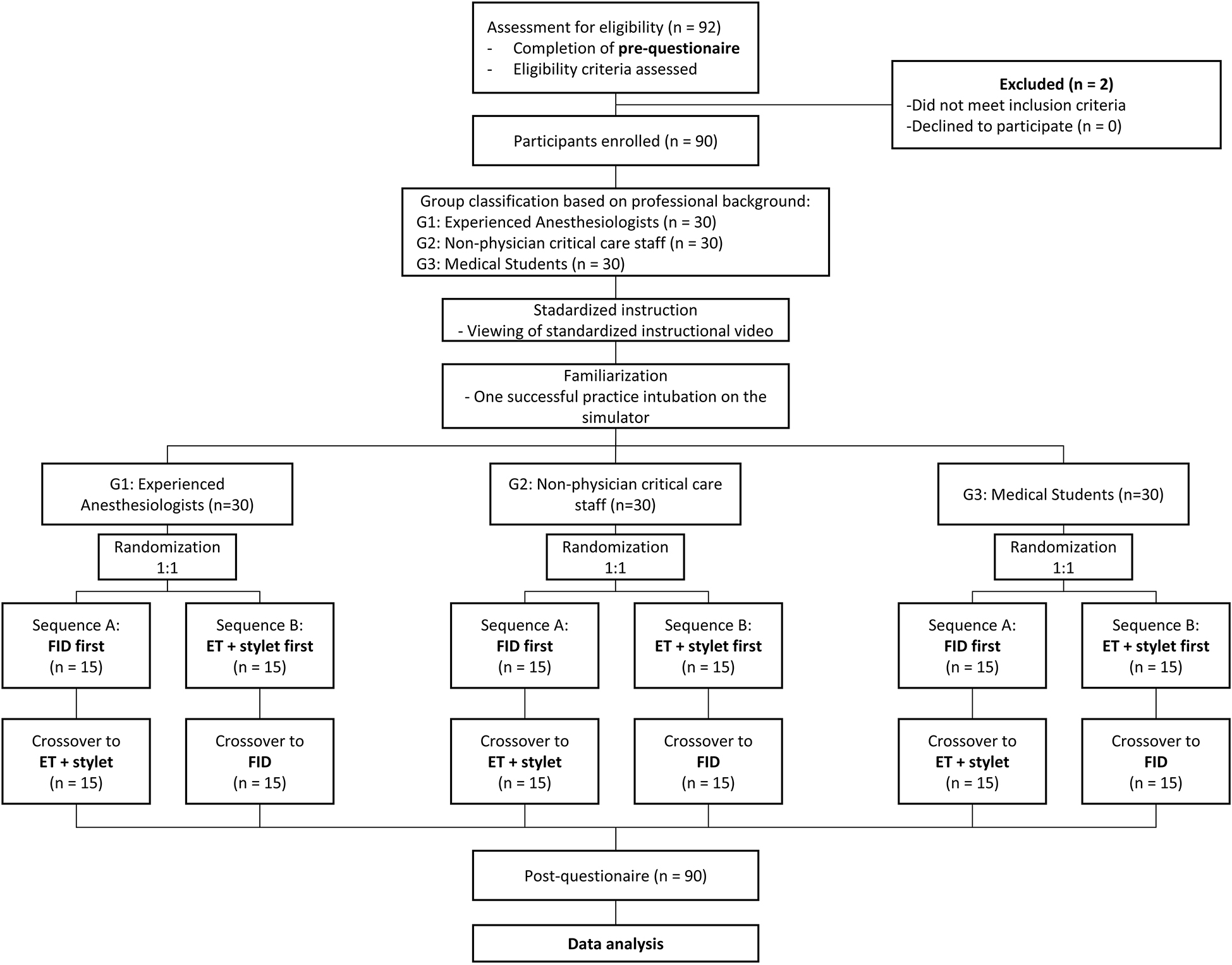
Study flow diagram showing eligibility assessment, group allocation, randomization, crossover, and follow-up

This dual-center, randomized crossover simulation study was conducted at the training facilities of the Departments of Anesthesiology, Intensive Care Medicine and Pain Therapy at University Hospital Frankfurt am Main and Sana Klinikum Offenbach am Main, Germany, between August 2023 and May 2025. All intubations were performed under controlled and standardized conditions using an easy-to-intubate airway manikin. The overall workflow (enrollment, group allocation, randomization, crossover, and follow-up) is shown in Fig. 1. Reporting follows established guidance for crossover randomized trials [11].

### Participants

Participants were recruited from predefined professional backgrounds relevant to airway management. Following completion of a standardized pre-study questionnaire (demographics, educational and professional background), participants were allocated to one of three predefined analysis groups:

- **Group 1 (G1)**: anesthesiologists, including anesthesiology residents in postgraduate year three or higher and board-certified anesthesiologists.
- **Group 2 (G2)**: non-physician critical care staff, including anesthesia nurses, anesthesia technicians, and registered nurses with at least two years of professional experience in anesthesiology, emergency medicine, or intensive care.
- **Group 3 (G3)**: medical students who had completed their curricular one-week anesthesiology and intensive care education block.

Participants whose background could not be clearly assigned were excluded from group-based analyses.

### Airway model and equipment

All intubations were performed using an airway management training manikin representing an easy-to-intubate scenario (Laerdal Airway Management Trainer, Stavanger, Norway) and a conventional direct laryngoscope with a Macintosh size 3 blade [12]. 

**Fig. 2:**
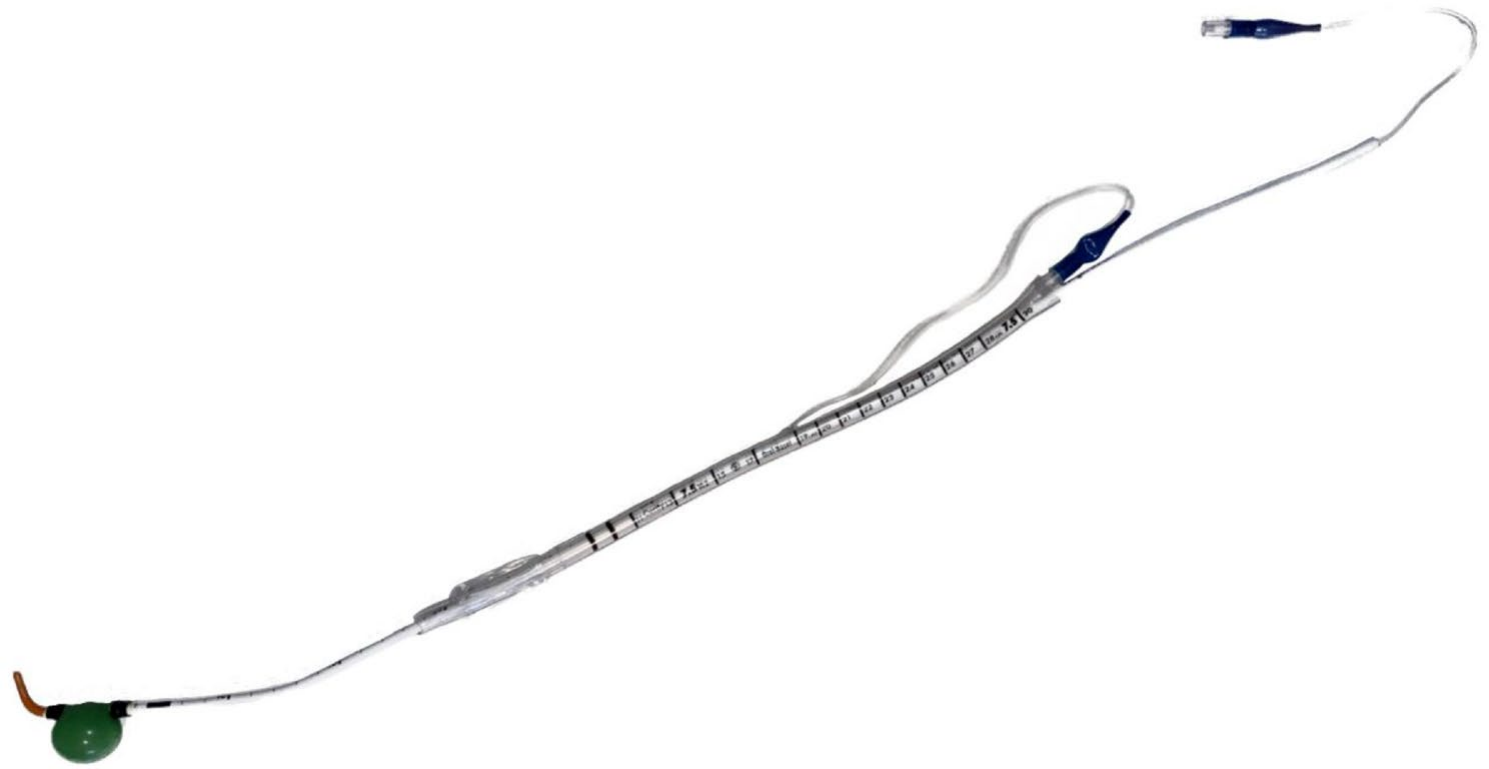
Photograph of the Frankfurt Intubation Device (FID) minimum viable product used in the present study. The prototype is based on a conventional intubation bougie (S-Guide, VBM Medizintechnik GmbH) and incorporates a distal inflatable balloon intended to provide early mechanical aspiration protection. Reproduced from Kloka et al. [10] under CC BY-NC 4.0

In the control condition, a standard endotracheal tube (Rusch^®^ Safety Clear^®^ Plus Murphy Eye Endotracheal Tube - Size 8.0) preloaded with a malleable stylet (Rusch^®^ Flexi-Slip™) was used. In the intervention condition, intubation was performed using the FID prototype, which is based on a conventional intubation bougie (S-Guide, VBM Medizintechnik GmbH) and incorporates a distal inflatable balloon intended to provide early mechanical aspiration protection, as previously described in our initial development and evaluation study (Fig. 2) [10]. The same type of endotracheal tube with a Murphy eye was used in both study arms.

### Experimental setup and procedure

After the pre-study questionnaire and group allocation, all participants received standardized instruction via a 3-minute instructional video covering the general intubation procedure, handling of the FID, and conventional intubation using an endotracheal tube with stylet.

Each participant then performed one successful familiarization intubation on the simulator with each device prior to data collection. Participants were randomized 1:1 by the study investigator to start with either the FID or the endotracheal tube with stylet and then crossed over to the alternative device (Fig. 1), using stratified sealed-envelope randomization to ensure allocation concealment. For each experience group, sealed opaque envelopes containing equal allocations to both starting devices were prepared in advance and drawn at random by the participants on the study day. This approach was used to balance potential training effects across the crossover sequence. A qualified assistant was available upon verbal instruction. Successful intubation was defined as visible lung inflation following manual ventilation of the manikin. No predefined maximum time limit was applied to an intubation attempt. In case of failed intubation, defined as absence of visible lung inflation (e.g. esophageal intubation), the attempt was classified as unsuccessful and the intubation sequence was restarted from the beginning with a new timing cycle. All time points were defined a priori using predefined procedural landmarks and were assessed based on video recordings. Tooth damage was defined as an audible clicking sound of the manikin’s upper teeth, indicating excessive pressure during laryngoscopy or tube advancement. All attempts were video recorded from a lateral perspective for precise time measurement and post-hoc review. In cases of ambiguity, video sequences were reviewed in full by the study investigators to determine the corresponding time point according to the predefined definitions. After both attempts, participants completed a post-study questionnaire assessing usability and perceived difficulty.

### Sample size

The sample size was planned to support a non-inferiority analysis of the primary time-based outcome in a paired crossover design. Sample size calculation was performed using Python (SciPy), assuming a one-sided α of 0.025, 80% power, and a non-inferiority margin of Δ = 5 s. Because no directly comparable prior data were available for the FID, a conservative standard deviation of 15 s for paired time differences was assumed based on published randomized airway studies with time-based procedural outcomes, including relevant non-inferiority studies [13, 14]. This yielded a required sample size of 71 participants. We therefore aimed to enroll 90 participants, allocating 30 participants to each predefined experience group to allow stable descriptive estimates across groups and to account for incomplete datasets. Subgroup analyses were exploratory and not powered for formal non-inferiority testing.

### Statistical analysis

The primary analysis assessed whether intubation using the FID was non-inferior to conventional ETI with stylet with respect to intubation time. The primary outcome was T2, defined as the time from insertion of the laryngoscope until inflation of a cuffed device positioned in the trachea (inflated endotracheal tube cuff in the control condition or inflated distal balloon of the FID). T2 was selected as the primary endpoint because it most directly reflects the intended procedural concept of the FID. A non-inferiority margin (Δ) of 5 s was chosen for the primary analysis. This margin is consistent with thresholds used in prior airway management studies assessing procedural time outcomes [14] and was selected to allow a conservative assessment of non-inferiority for this novel device. The mean paired difference in T2 (FID minus ET+stylet) and its two-sided 95% confidence interval (CI) were calculated using a paired, mean-based approach. No formal normality test was performed. Although time-based outcomes may be skewed, inference was based on the sampling distribution of the mean paired difference. As a sensitivity analysis, 95% CIs were additionally obtained using non-parametric bootstrap resampling; results were consistent. Non-inferiority was concluded if the upper bound of the CI did not exceed Δ.

Secondary outcomes included DT2 (time from view of the glottis until cuffed device positioned in the trachea) and T3 (time from insertion of the laryngoscope until ventilation), analyzed descriptively using the same paired analytical approach. Binary secondary outcomes included first-pass success and tooth damage and were analyzed as paired risk differences with corresponding 95% CIs.

Exploratory subgroup analyses were performed for predefined experience groups (G1–G3) and by randomization sequence to assess consistency. Exploratory analysis of training effects was performed using paired visualization of T1 across the first (Run 1) and second intubation attempt (Run 2). T1, defined as the time from laryngoscope insertion to glottic visualization, was chosen as a device-independent measure of laryngoscopy performance and the parameter most likely to reflect simulator-based learning between repeated attempts. These analyses were descriptive and not powered for formal non-inferiority testing. All analyses were performed using Python (version 3.11.5). Non-inferiority conclusions were based exclusively on confidence-interval assessment.

## Results

### Participants

**Table 1:** Participant characteristics and group allocation

	Overall	Experienced Anesthesiologists [G1]	Non-physician critical care staff [G2]	Medical students [G3]
Participants, *n*	90	30	30	30
Age, mean (SD)	32.9 (9.9)	37.3 (8.9)	36.1 (9.4)	25.2 (6.2)
Sex: Female, n (%)	46 (51.1%)	15 (50.0%)	14 (46.7%)	17 (56.7%)
Sex: Male, n (%)	42 (46.7%)	14 (46.7%)	16 (53.3%)	12 (40.0%)
Sex: Not Reported, n (%)	1 (1.1%)	1 (3.3%)	0 (0.0%)	0 (0.0%)
Sex: Diverse, n (%)	1 (1.1%)	0 (0.0%)	0 (0.0%)	1 (3.3%)
Sequence A: FID first, n (%)	45 (50.0%)	-	-	-
Sequence B: ET+stylet first, n (%)	45 (50.0%)	-	-	-

Ninety participants completed the study and were included in the analysis (30 per experience group). Participant characteristics and group allocation are summarized in Table 1. All participants performed both intubation attempts according to the randomized crossover design, resulting in 90 paired observations for all outcomes.

### Primary outcome (T2)

**Fig. 3:**
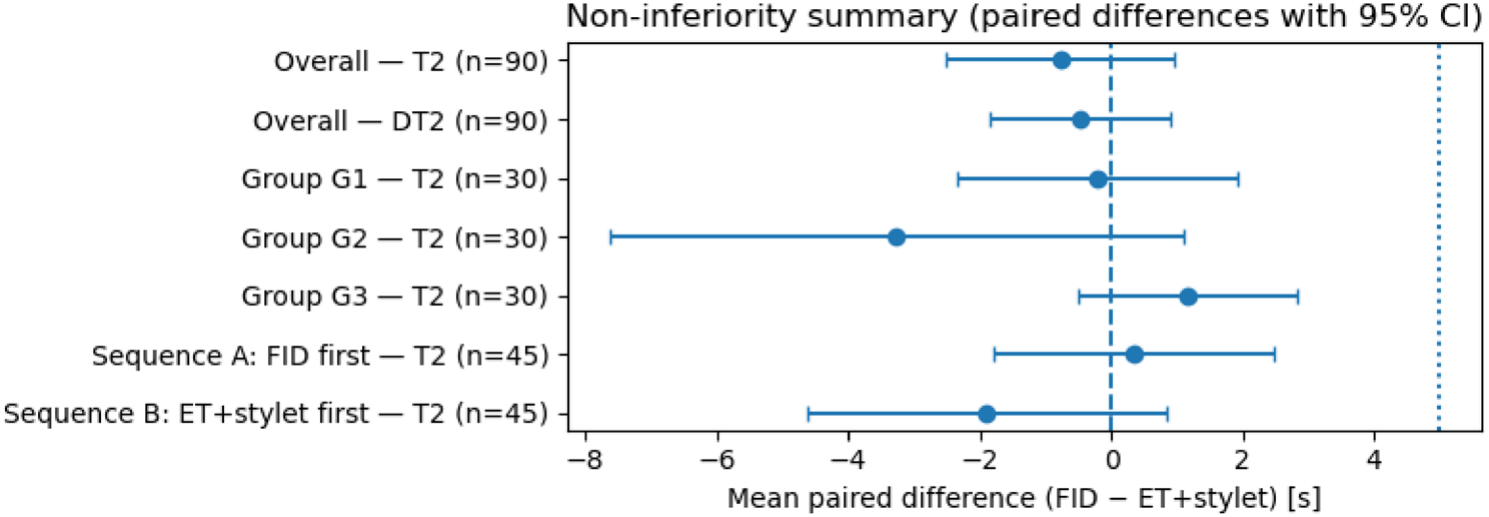
Non-inferiority summary plot showing mean paired differences (FID − ET+stylet) with 95% confidence intervals for T2 overall and in exploratory subgroup and sequence analyses; the vertical dotted line indicates the predefined non-inferiority margin (Δ = 5)

Across all participants, the mean paired difference in T2 (FID − ET+stylet) was − 0.77 s (95% CI − 2.50 to 0.97 s), meeting the predefined non-inferiority criterion (Δ = 5 s). A non-inferiority summary is shown in Fig. 3, and numeric results are provided in Table 2. As an exploratory diagnostic analysis, Bland–Altman plots were used to visualize paired differences in T2 across the measurement range; no relevant systematic bias was observed (Supplementary Figure S1).

**Table 2:** Primary and secondary outcomes from paired comparisons of FID and ET+stylet

	FID mean	ET+stylet mean	Paired diff (FID − ET)	95% CI (diff)	NI margin Δ (s)	Non-inferior?
Time to airway protection [T2] (primary) (s)	13.27	14.03	-0.77	[-2.50, 0.97]	5	True
*Glottic view to airway protection* [DT2] (s)	7.94	8.42	-0.48	[-1.85, 0.89]	5	True*
*Time to ventilation* [T3] (s)	33.66	19.58	14.08	[12.03, 16.13]	5	False*
First-pass success (%)	96.67	94.44	2.22	[-3.1, 7.6]	-	-
Tooth damage (%)	7.78	14.44	-6.67	[-12.7, -0.6]	-	-

Primary and secondary outcomes comparing the Frankfurt Intubation Device (FID) with ET + stylet (paired crossover analysis), * Non-inferiority for DT2 and T3 is reported descriptively (secondary outcome)

### Secondary time-based outcomes

For DT2, the mean paired difference was − 0.48 s (95% CI − 1.85 to 0.89 s). For T3, the mean paired difference was 14.08 s (95% CI 12.03 to 16.13 s). Secondary outcomes are summarized in Table 2.

### Subgroup and sequence analyses

Exploratory analyses demonstrated consistent findings for T2 across experience groups (G1–G3) and randomization sequences. Detailed subgroup and sequence results are provided in Supplementary Table S2, with an overview shown in Fig. 3.

### Binary outcomes

First-pass success was achieved in 96.7% of FID attempts and 94.4% of ET+stylet attempts (paired risk difference + 2.2% points, 95% CI − 3.1 to 7.6). Tooth damage occurred in 7.8% of FID attempts and 14.4% of ET+stylet attempts (paired risk difference − 6.7% points, 95% CI − 12.7 to − 0.6). Full results are reported in Table 2.

**Fig. 4:**
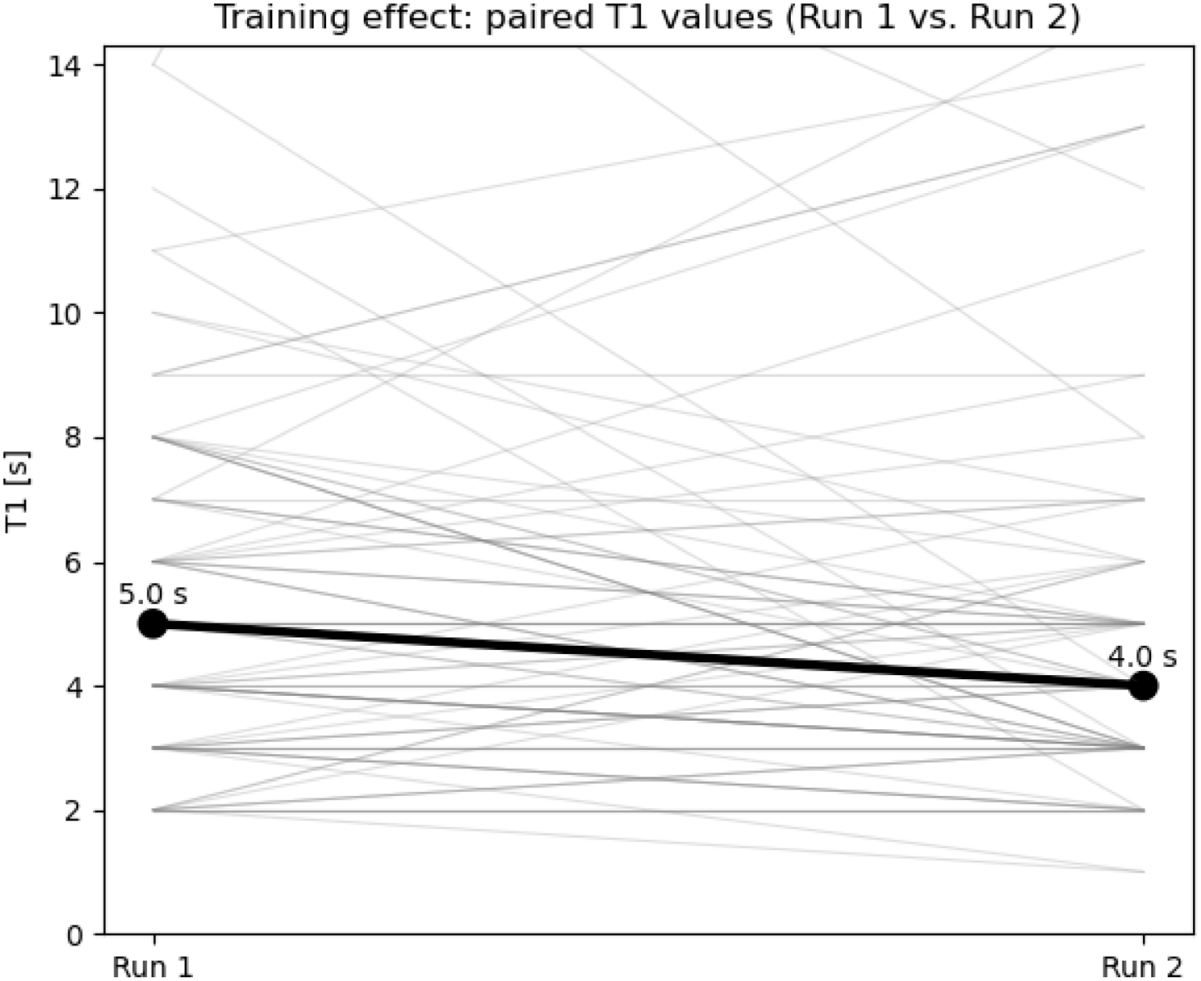
Training effect on T1. Paired T1 values for the first (Run 1) and second (Run 2) intubation attempt are shown for all participants. Gray lines represent individual paired values; black points and line indicate the median. For visualization purposes, the y-axis was limited to the 95th percentile of T1 values to improve readability; a small number of higher values are therefore clipped

An exploratory analysis demonstrated a training effect between the first and second intubation attempt, independent of the device used. Paired visualization of T1 showed shorter times in the second attempt compared with the first (Fig. 4). Median T1 decreased from 5.0 s in Run 1 to 4.0 s in Run 2.

## Discussion

In this dual-center randomized crossover simulation study using an easy-to-intubate airway model, the Frankfurt Intubation Device (FID) achieved non-inferior time to securing the airway and providing early mechanical protection against aspiration (T2) compared with standard endotracheal intubation using an endotracheal tube with stylet. While first-pass success provided the broader clinical rationale for the study, the primary endpoint was predefined as time to airway protection (T2), because this most directly captures the intended combination of bougie-guided tracheal access and early mechanical aspiration protection. The paired T2 differences showed moderate variability without relevant systematic bias, and the non-inferiority conclusion was robust, as reflected by confidence intervals clearly below the predefined margin and confirmed by bootstrap sensitivity analyses. As anticipated, a training effect was observed on the simulator. This effect was assessed across the full cohort; although a stronger simulator-based learning effect in the less experienced groups would be plausible, the study was not designed to formally compare training effects between predefined experience groups. The balanced 1:1 randomization of the starting device ensured that this learning effect was evenly distributed across study arms, thereby limiting its impact on the paired device comparison. First-pass success was high for both techniques, while tooth damage occurred less frequently with the FID.

A secondary finding was a longer time to ventilation (T3) with the FID. While this difference is likely clinically relevant, the present study was designed to assess baseline performance under standardized conditions. The FID represents a novel device, and familiarization time was short, which may have contributed to longer ventilation times (T3) during early use. In addition, workflow efficiency may improve with further user training and with refinement of the current prototype toward an industrially manufactured device optimized for clinical use. In more challenging airway scenarios, time to ventilation with conventional ETI may increase as first-pass success decreases and repeated attempts become necessary [2, 3, 6]. Nevertheless, reducing time to ventilation remains a key objective for future device iterations.

The FID was designed to support users who may need to perform emergency intubation under pressure and under suboptimal laryngoscopy conditions. Current airway guidelines appropriately favor experienced operators for difficult or emergency airways whenever possible [4]. However, emergency airway management in real-world prehospital and critical care settings remains dependent on operator, team, and system factors, and operator experience is consistently associated with improved first-pass success. In this context, the staged evaluation of devices intended to simplify airway management in demanding settings remains clinically relevant [15, 16]. The FID should be viewed as an innovative device undergoing staged evaluation rather than simply as another addition to the airway device arsenal. Its intended role is to combine bougie-guided tracheal access with early mechanical aspiration protection, particularly in difficult-airway and time-critical scenarios, which were intentionally not yet modeled in this standardized proof-of-concept study. Although this study focused on an easy-airway model, the pattern observed in secondary outcomes is consistent with the rationale underlying bougie-guided techniques, which can facilitate tracheal access in restricted glottic views [7–9]. The earlier development and usability evaluation of the FID supports the feasibility of this approach in low-experience operators [10]. These findings are hypothesis-generating and support further evaluation in difficult-airway and time-critical simulation settings, before clinical implementation [5, 17]. 

The observed reduction in tooth damage may reflect differences in laryngoscopic workflow and tube advancement between both techniques. Qualitatively, the FID appeared easier to advance into the trachea even when glottic exposure was not optimal, whereas with the endotracheal tube and stylet, tube placement sometimes appeared more difficult despite visualization of the vocal cords. However, this study was not designed to measure applied force during laryngoscopy. This potential safety signal warrants further investigation. During execution of the study protocol, we observed occasional workflow- and handling-related challenges rather than device failure, including difficulties advancing the endotracheal tube over the FID. This appeared to be attributable primarily to premature removal of the laryngoscope before tube advancement, which promoted interaction with laryngeal structures, rather than to insufficient lubrication. Additional handling issues included excessive force or incomplete balloon deflation before withdrawal of the FID. These observations underscore the importance of structured training and iterative refinement during early device evaluation and provide practical input for further optimization of handling and device design. Further industrial collaboration may facilitate continued refinement toward a patient-ready device.

From an implementation perspective, this study represents a step within a staged evaluation pathway. Future work should prioritize iterative refinement aimed at reducing time to ventilation, followed by simulation studies in more complex airway scenarios, including limited glottic view, soiled airway conditions, and under increased time pressure.

### Limitations

Intubations were performed on an easy-to-intubate airway manikin under controlled conditions. These results may not generalize to difficult airway anatomy, soiled airways, or the physiological instability and time pressure typical for emergency intubation. A qualified assistant familiar with the study workflow was available; in clinical practice, assistant experience and familiarity with the device may vary and could influence performance, particularly time to ventilation. Manikin-based outcomes are proxies and cannot capture patient-centered endpoints such as hypoxemia, aspiration events, hemodynamic responses, or airway trauma. Finally, despite randomized crossover allocation, learning and carryover effects are possible in procedural simulation studies, and subgroup analyses were exploratory and not powered for formal non-inferiority conclusions. Group 1 also comprised anesthesiology residents and board-certified anesthesiologists and was therefore not fully homogeneous with respect to airway experience. Although this is less likely to have materially affected the primary paired comparison, it should be considered when interpreting group-level findings.

## Conclusions

In this dual-center randomized crossover simulation study using an easy-to-intubate airway model, the Frankfurt Intubation Device (FID) demonstrated non-inferior time to securing the airway with early mechanical protection against aspiration compared with standard endotracheal intubation using an endotracheal tube with stylet. Secondary findings suggest potential safety-related advantages, while time to ventilation remains an important target for further optimization. These results support continued staged evaluation of the FID, particularly in more challenging airway scenarios and in users with limited airway experience.

## Supplementary Information

Below is the link to the electronic supplementary material.

Supplementary Material 1

Supplementary Material 2

## Data Availability

The datasets generated and/or analyzed during the current study are not publicly available, as they contain detailed individual-level performance data from simulation participants and were collected under consent that did not include unrestricted public data sharing. The data are available from the corresponding author on reasonable request.

## Abbreviations

CI: Confidence interval
DT2: Time from visualization of the glottis to airway protection
ET: Endotracheal tube
ETI: Endotracheal Intubation
ET+stylet: Endotracheal tube with stylet
FID: Frankfurt Intubation Device
FPS: First–pass intubation success
NI: Non–inferiority
SD: Standard deviation
T1: Time from laryngoscope insertion to visualization of the glottis
T2: Time from laryngoscope insertion to airway protection
T3: Time from laryngoscope insertion to first effective ventilation
Δ: Non–inferiority margin
