# Supplementary figures and images for: Time to intubate with an innovative intubation device: a dual-center randomized crossover non-inferiority simulation study
Source: Int J Emerg Med. 2026 Apr 11;19:98. doi: 10.1186/s12245-026-01208-y (PMC13077913; doi:10.1186/s12245-026-01208-y)

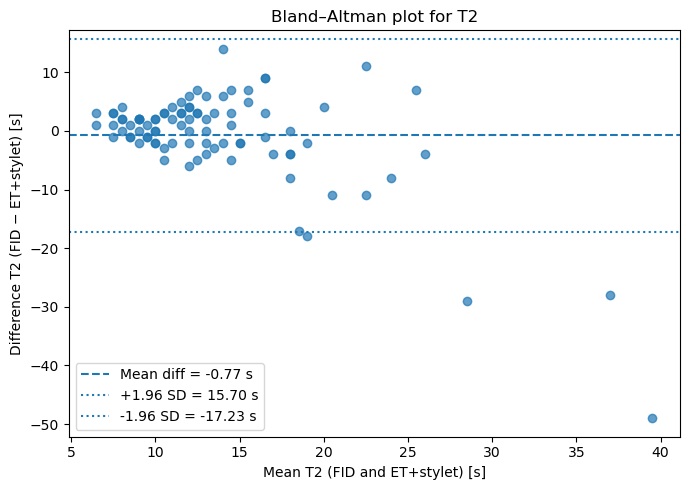

Supplement: Supplementary file 1 — Supplementary Material 1 [file 12245_2026_1208_MOESM1_ESM.jpg]
